# Correlation Between MRI Characteristic of Osteosarcoma with 2-Year Survival Outcomes

**DOI:** 10.3390/diagnostics15131707

**Published:** 2025-07-03

**Authors:** Mohd Noor Akmal Adam, Emilia Rosniza Mohammed Rusli, Erica Yee Hing, Juliana Fairuz Maktar, Ckhai Loh, Nor Hazla Mohamed Haflah, Faizah Mohd Zaki

**Affiliations:** 1 Department of Radiology, Faculty of Medicine, Universiti Kebangsaan Malaysia, Cheras Kuala Lumpur 56000, Malaysia; p109682@siswa.ukm.edu.my (M.N.A.A.); emilia.rosniza.hctm@ukm.edu.my (E.R.M.R.); ericayeehing@hctm.ukm.edu.my (E.Y.H.); julianafairuz@yahoo.com (J.F.M.); 2 Department of Pediatrics, Faculty of Medicine, Universiti Kebangsaan Malaysia, Cheras Kuala Lumpur 56000, Malaysia; ckhailoh@hctm.ukm.edu.my; 3 Department of Orthopaedics, Faculty of Medicine, Universiti Kebangsaan Malaysia, Cheras Kuala Lumpur 56000, Malaysia; norhazla@hctm.ukm.my

**Keywords:** MRI, osteosarcoma, 2-year survival

## Abstract

**Background:** Magnetic resonance imaging (MRI) plays a crucial role in staging and preoperative evaluation in osteosarcoma patient. Fewer studies have focused on 2-year survival, which reflects tumour aggressiveness and early disease progression. This study examines the association between MRI characteristics and 2-year survival outcomes in osteosarcoma to better understand the imaging characteristic of high-risk patients. **Methods:** A retrospective case–control study was conducted at a tertiary university hospital. Patients diagnosed with osteosarcoma between 2010 and 2022 were included if they had a pre-treatment MRI and at least 2 years of follow-up. MRI scans were reviewed by two blinded radiologists to assess tumour location, volume, growth pattern, presence of fluid–fluid levels (FFL), pathological fractures, skip metastases, neurovascular bundle involvement, regional lymphadenopathy, and physeal or joint involvement. Statistical analyses, including Fisher’s exact test, Chi-square test, and Mann–Whitney U test, were performed to determine associations between MRI features and survival outcomes. **Results:** Twenty-eight patients (*n* = 28) met the inclusion criteria. Larger tumour volume (>300 mls) was significantly associated with poorer 2-year survival (*p* = 0.008). The presence of skip metastases also correlated with worse outcomes (*p* = 0.041). While presence of FFL, concentric growth pattern, regional lymphadenopathy, and physeal involvement showed trends toward poorer prognosis, these associations were not statistically significant. **Conclusions:** MRI characteristics, particularly tumour volume and skip metastases, are significant prognostic indicators of 2-year survival in osteosarcoma. These findings highlight the potential role of MRI in risk stratification and treatment planning, aiding in the identification of high-risk patients that can help with management.

## 1. Introduction

Osteosarcoma is a malignant bone cancer derived from primitive bone-forming mesenchymal cells, characterized by excessive production of osteoid or immature bone. It is the most common primary bone cancer, predominantly affecting children and adolescents. Worldwide, the annual incidence of osteosarcoma is 3.4 cases per million people and 5.6 cases per million children under the age of 15 [1]. In Malaysia, bone cancer is the fourth most common cancer in childhood and adolescence. The incidence rate of osteosarcoma in Malaysia between 2012 and 2016 was much lower, at 0.65 per million for males and 0.79 per million for females under 14 years old [2].

The survival rate of patients with osteosarcoma has significantly improved since the 1970s with the introduction of better neoadjuvant chemotherapy regimens. The current 5-year survival rate is approximately 60% to 80%. Survival outcomes are multifactorial, influenced by age at presentation, tumour size or stage, tumour location, presence of metastasis, and response to preoperative chemotherapy. A 2024 review article highlighted that the introduction of neoadjuvant chemotherapy in the 1970s, specifically the MAP regimen (methotrexate, doxorubicin, and cisplatin), significantly improved survival rates where the 5-year survival rate is approximately 65% for patients with localized disease, but it drops dramatically to 10–20% in cases with metastatic disease. Despite advancements, the development of better therapeutic approaches has stalled, creating a plateau in patient outcomes that has persisted for 40 years [3,4].

Magnetic resonance imaging (MRI) is the imaging modality of choice for staging and preoperative evaluation of osteosarcoma. It is the preferred cross-sectional imaging technique due to its robust function to assess soft tissue, joint, and neurovascular bundle invasion, as well as to identify skip metastases. Tumour volume, location, morphology, neurovascular bundle involvement, and metastases have been shown to influence prognosis and predict response to neoadjuvant chemotherapy, which subsequently affects overall survival [5].

Numerous studies have described clinical and imaging features that affect prognosis in osteosarcoma, with MRI playing a crucial role in assessing tumour characterisation and local involvement. The 5-year survival rate is commonly used in most studies as it provides a more comprehensive long-term outcome measure. Faisham et al. reported that the survival rate for osteosarcoma patients who completed chemotherapy and surgery was 72% at 2 years but dropped to 44% at 5 years [6]. While the 5-year survival rate is the conventional benchmark for long-term prognosis in osteosarcoma and serves as a standard timeframe for future comparative studies, the 2-year survival rate provides crucial insight into disease aggressiveness. A comprehensive analysis by the Children’s Oncology Group revealed that approximately 30% of patients with localized osteosarcoma experience a recurrence, with the majority occurring within 2 years of initial treatment [7].

However, the correlation between MRI characteristics and 2-year survival outcomes has not been thoroughly explored from a radiological perspective. Given that MRI is the preferred imaging modality for osteosarcoma evaluation, it is essential for radiologists to accurately identify key imaging that correlates with poorer 2-year survival. This could help stratify patients who may benefit from more intensive treatment strategies rather than standard protocols. Additionally, a 2-year survival metric allows for earlier assessment of treatment efficacy, guiding adjustments in therapeutic approaches and follow-up intensity. This is particularly important for risk-adaptive therapy and personalized treatment planning, ensuring that high-risk patients receive timely, targeted interventions and informed family counselling.

To the best of our knowledge, no study has examined the correlation between MRI features and 2-year survival outcomes in osteosarcoma in Malaysia. Most studies on imaging prognostic factors are based on overseas data and may not fully represent outcomes in our local population. Thus, this study aims to enhance the understanding of MRI-based prognostic indicators and their correlation with 2-year survival rate, facilitating clinical decision-making, especially in a Malaysian population.

## 2. Materials and Methods

### 2.1. Patient Selection

This was a retrospective study conducted in the Radiology Department of a tertiary university hospital. A list of patients with osteosarcoma from 2010 to 2022 was collected from the International Centre for Casemix and Clinical Coding (ITCC) unit as well as from the census records of the Orthopaedic Oncology and Paediatric Oncology clinics. Data collected included patient’s age, gender, and race.

This study was conducted in accordance with the Declaration of Helsinki, and the protocol was approved by the Ethics Committee of Faculty of Medicine Universiti Kebangsaan Malaysia (Project identification code: FF-2022-174) on 22 June 2022. Informed written consent of the subject was waived in view of the retrospective nature of the study.

The inclusion criteria included patients who had been diagnosed with primary conventional osteosarcoma and were under follow-up in our hospital for at least two years from the date of diagnosis; had undergone treatment—either chemotherapy, surgical intervention, or a combination of both; and had undergone a pre-treatment MRI that was available for secondary review. Any patient who did not fulfil all of the criteria were excluded from the study.

### 2.2. Analysis and Definition of Study Variable

MRI studies (20 out of 28) were performed following protocols from either of the two different machines available in the institution; Siemens Avanto 1.5T (Erlangen, Germany) and Siemens Verio 3.0T (Erlangen, Germany) with bone tumour protocol MRI sequences including coronal T1, STIR and T1 fat saturation post-gadolinium, axial T1, T1 fat saturation, T2 fat saturation and T1 fat saturation post-gadolinium as well as sagittal T2 and T1 fat saturation post-gadolinium (Table 1). DWI/ADC was only performed in recent years and thus was not included in the final MRI parameter assessed. Eight MRI studies were performed in different healthcare centres.

The MRI studies were reviewed jointly by two experienced radiologists (a paediatric radiologist of 14 years’ experience and a musculoskeletal radiologist of 7 years’ experience), both blinded to the histology findings and patient outcomes. Images were reviewed using the DICOM viewer platform (Horos^®^). Specific MRI imaging features were assessed in each MRI study, including tumour location, volume, and growth pattern, as well as the presence of fluid–fluid levels (FFL) within the tumour. The presence or absence of pathological fractures, skip metastases, neurovascular bundle involvement, regional lymphadenopathy, as well as physeal and joint involvement were also assessed. Any discrepancy was resolved by consensus between the readers and thus interobserver variability assessment was not performed.

Tumour location was determined based on the primary site of the tumour, categorized as humerus, tibia, femur, fibula, or ilium.

Tumour volume of the primary tumour was calculated using the formula: 0.52 × tumour depth × tumour width × tumour length [6]. Skip metastasis, if present, were not included or added in the tumour volume.

**Figure 1:**
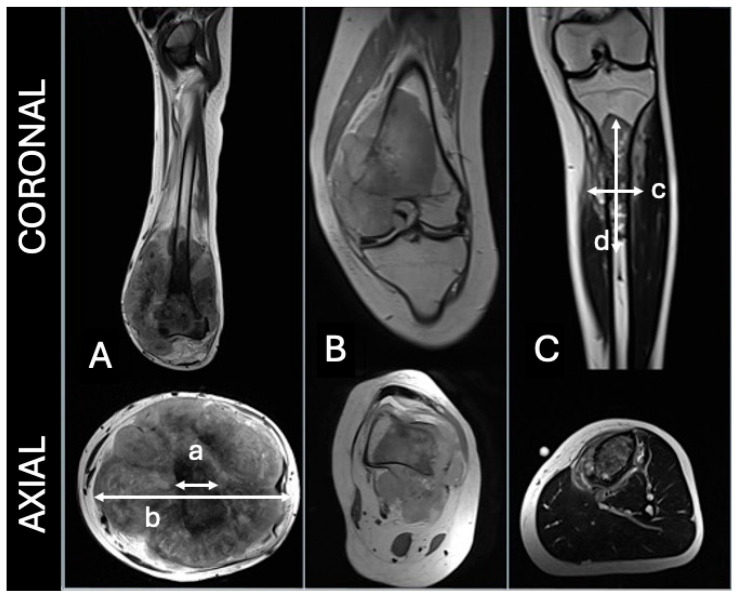
(**A**)—concentric, (**B**)—eccentric, (**C**)—longitudinal. For concentric, the tumoral width should be 1.5 greater than bone diameter (b/a > 1.5). For longitudinal, tumoral length should be 2 times greater than width (d/c > 2). Adapted from Kim et al. [8].

Tumour growth pattern was classified according to the criteria established by Kim et al. [8] into three categories: concentric, eccentric, or longitudinal (Figure 1).

Skip metastasis was defined as tumour foci separated from the primary tumour by normal medullary bone, or in the adjacent bone (trans-articular skip metastasis), and was recorded as either present or absent.

**Figure 2:**
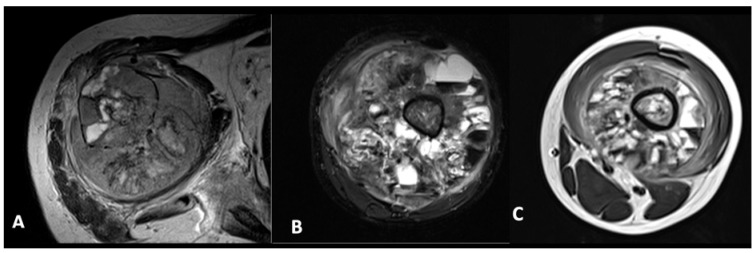
Features of FFL within tumour. (**A**)—less than one-third. (**B**)—between one-third to two-thirds. (**C**)—more than two-thirds.

Fluid–fluid level (FFL) within the tumour was identified based on the presence of varying signal intensities in the T2-weighted (T2W) sequence and further categorized according to the classification by Jeon et al. (Figure 2) [9]:

- FFL occupying less than one-third of the lesion;
- FFL occupying between one-third and two-thirds of the lesion;
- FFL occupying more than two-thirds of the lesion.

Regional lymphadenopathy was defined as the presence of suspicious lymph nodes (thickened cortex, loss of fatty hilum, size more than 1 cm or presence of mineralisation/calcification) adjacent to the tumour site (axilla in upper limb; inguinal and popliteal in lower limb).

The 2-year survival outcome was classified into two groups: good outcome—disease-free survival with no recurrence within 2 years; poor outcome—tumour progression, recurrence, or death within 2 years.

### 2.3. Statistical Analysis

Fisher’s exact and Chi-square test were used to identify correlation between each MRI’s features with survival outcome. Mann–Whitney U test was used for correlation between tumour volume (numerical) with survival outcome. *p* values of <0.05 were considered significant. Statistical analysis was performed using SPSS version 30.0.

## 3. Results

### 3.1. Statistical Analysis

**Figure 3:**
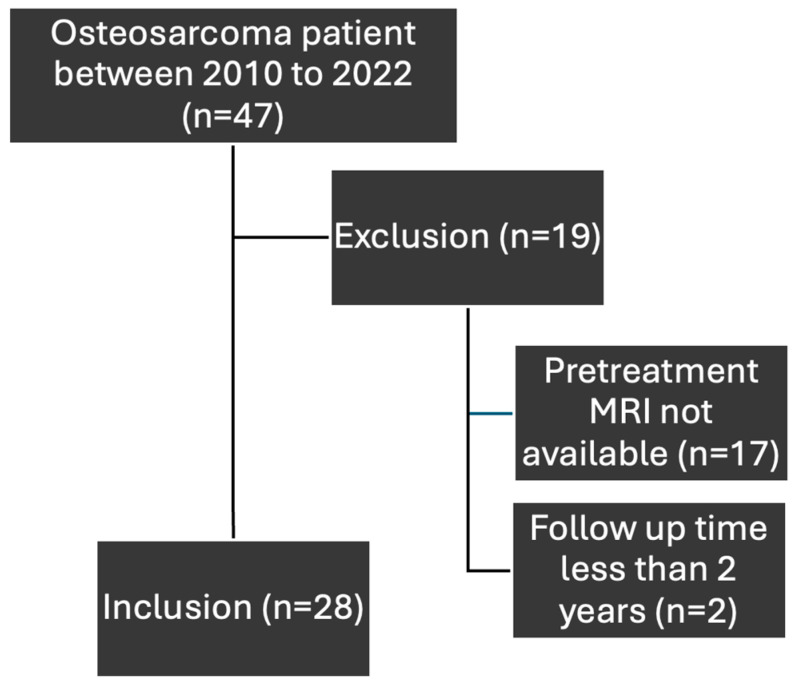
Flowchart for inclusion and exclusion of osteosarcoma patients.

Between 2010 and 2022, 47 patients with osteosarcoma were managed at our institution and were identified as the sample population. However, only 28 patients were included in this study as >50% of them met exclusion criteria. The flowchart of inclusion and exclusion of osteosarcoma patients is shown in Figure 3. Their baseline characteristics are shown in Table 2.

### 3.2. MRI Characteristics Evaluation of the Sample Cohort

**Figure 4:**
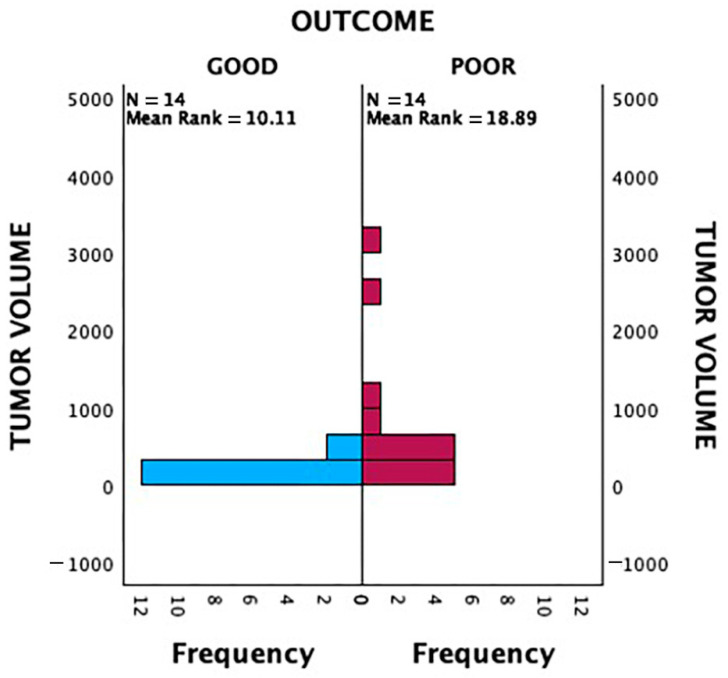
Independent-samples Mann–Whitney U Test analysis of tumour volume and patient outcome (*p* < 0.05).

A Mann–Whitney U test was conducted to evaluate the association between tumour volume on MRI and 2-year survival outcomes (Figure 4). Patients with poor outcomes had significantly larger tumour volumes compared to those with good outcomes, indicating that tumour volume is a significant prognostic factor (*p* = 0.004).

**Figure 5:**
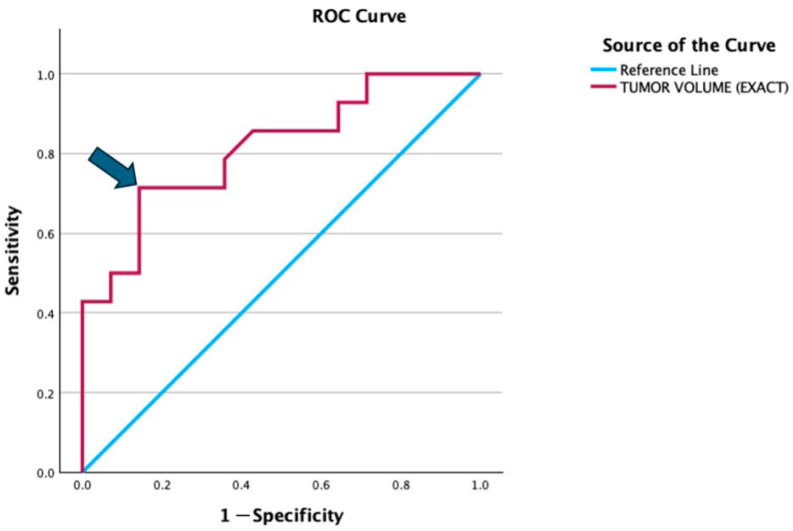
ROC curve illustrates the diagnostic performance of tumor volume in distinguishing between the good and poor prognosis groups. The red line represents the ROC curve for tumor volume, while the blue diagonal line is the reference line indicating no discrimination (AUC = 0.5). The arrow highlights the optimal cut-off point (322.5 mls), which corresponds to a sensitivity of 71.4% and specificity of 85.7% (1 − specificity = 0.143).

We performed receiver operating characteristic (ROC) curves to determine the cut-off value of tumour volume (Figure 5). It showed the cut-off value was approximately 322.5 mls for tumor volume with a sensitivity of 71.4% and specificity of 85.7%. Thus, we decided to take 300 mls tumour volume as the cut-off value for the outcome; between good or poor prognosis.

**Figure 6:**
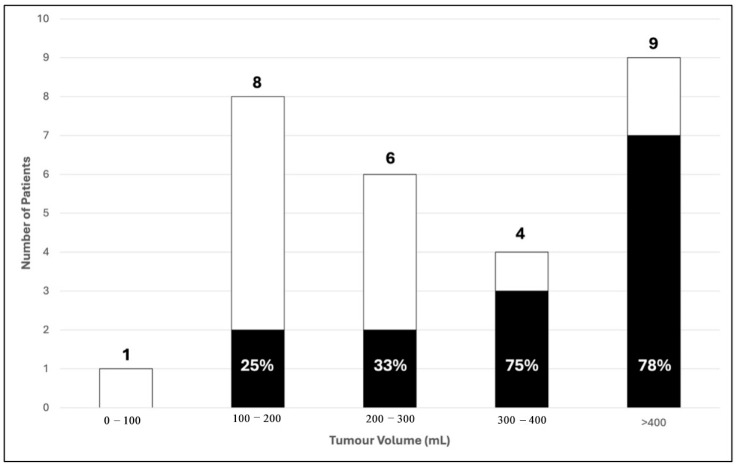
Distribution of tumour volume with 2-year survival outcome in 28 patients. (■) Poor; (☐) Good 2-year survival outcome.

In total, 2 out of 8 patients (25%) with a tumour volume between 100 and 200 mls and 2 out of 6 patients (33%) with a tumour volume between 200 and 300 mls had poor 2-year prognostic outcome. For patients with a tumour volume between 300 and 400 mls, there was significant increase in the proportion of patients with poor 2-year prognosis with 75% (3 out of 4). This percentage does not increase further in patients with tumour volume more than 400 mls (Figure 6). Therefore, we categorised our data with 300 mls as a critical value for comparative analysis. Among patients with tumour volumes < 300 mls, 73.3% (11/15) had good outcomes, while 26.7% (4/15) had poor outcomes. Conversely, for patients with tumour volumes > 300 mls, only 23.1% (3/13) achieved good outcomes, whereas the majority (76.9%, 10/13) had poor outcomes. This association was found to be statistically significant (*p* = 0.008) (Table 3).

Among the three tumour growth patterns (concentric, eccentric, and longitudinal), concentric tumours had the highest proportion of poor outcomes (70%). Even when eccentric and longitudinal tumours were grouped, 61.1% had a good outcome compared to only 30.0% in the concentric group. However, these associations were not statistically significant (*p* = 0.258 for individual patterns, *p* = 0.115 for the grouped analysis) (Table 3).

No statistically significant correlation was observed between tumour location and outcome (*p* = 0.310). It is worth noting that in this study, humeral tumours had the most favourable prognosis, with 100.0% of patients (*n* = 3) achieving a good outcome. In contrast, iliac tumours had the worst prognosis, with 100.0% of patients (*n* = 1) experiencing a poor outcome (Table 3).

The presence of skip metastases was significantly associated with poorer clinical outcomes (*p* = 0.041). Among patients with skip metastases, 100% (5/5) experienced a poor outcome, with no cases of good outcome observed. In contrast, in the absence of skip metastases, 60.9% (14/23) of patients had a good outcome, while 39.1% (9/23) had a poor outcome.

The presence of FFL is associated with poorer survival outcomes, as suggested by the lower survival rate among patients with FFL (33.3% vs. 69.2%), however, this was not found to be clinically significant. The extent of FFL within the tumour also does not appear to significantly impact prognosis (Table 4).

Although not statistically significant, the presence regional lymphadenopathy, involvement of the neurovascular bundle, as well as physeal involvement, are associated with a poorer prognosis (Table 4). Nevertheless, these non-statistically significant findings have to be interpreted with caution.

No correlation was found between 2-year survival outcome with pathological fracture as well as joint involvement.

The Kappa value between reader 1 and reader 2 was 0.7 which is substantial.

## 4. Discussion

Both locally and globally, osteosarcoma is more common in males than females, with a peak incidence in the second decade of life [6,10]. This trend was reflected in this study, where significantly more males were affected than females, with a mean age of 17 years old (Table 2). Osteosarcoma usually occurs in the teenage years, aligning with the pubertal growth spurt. A comprehensive analysis of the SEER (Surveillance, Epidemiology, and End Results) database from 1975 to 2017 revealed that osteosarcoma is more common in males than females, with a peak incidence in the second decade of life. Specifically, the male-to-female ratio was 1.3:1, and the highest incidence occurred in the 15–19-year age group [3].

Larger tumour (>300 mls) and presence of skip metastases were found to be significantly correlated with poorer 2-year outcomes in our patients. We selected 300 mls as the cut-off point as it showed significant changes towards poorer 2-year survival outcome as depicted in Figure 4 and based on the ROC curve in Figure 5. This is consistent with multiple studies in the past. A study from Romania reported that a significant proportion of patients presented with tumors larger than 10 cm, which may reflect delayed diagnosis, potentially affecting the prognostic significance of tumor size [10]. Another study found that tumor volume greater than 200 mL was significantly associated with decreased survival rates in pediatric osteosarcoma patients [11].

Similarly, a retrospective analysis of 61 patients found a strong association between larger tumours and skeletal metastases, further contributing to poorer overall survival. Patients with tumours ≥ 1380 cm^3^ were 13.6-times more likely to have skeletal metastases (OR = 13.6, *p* < 0.01) [12]. Another study in India involving 825 patients reinforced these finding by demonstrating a tumour of >8 cm had significantly affected overall survival. Although they included only high-grade osteosarcomas treated with curative intent, the correlation between tumour size and overall survival remains a crucial finding that should be emphasized [13].

There are many theories on the reason behind poorer outcome in larger tumours, one of them being that a large tumour has a bigger hypoxic area in the centre. The hypoxic tumour microenvironment leads to drug and radiation resistance which significantly affect clinical outcomes. This has been a growing area of interest to specifically target hypoxia in cancer therapy [14,15]. While osteosarcomas are generally responsive to chemotherapy, surgery remains essential for curative treatment. Larger osteosarcomas also make complete surgical resection more difficult due to involvement with surrounding structures, increasing the risk of positive margins and local recurrence and significantly affects long-term survival [16].

**Figure 7:**
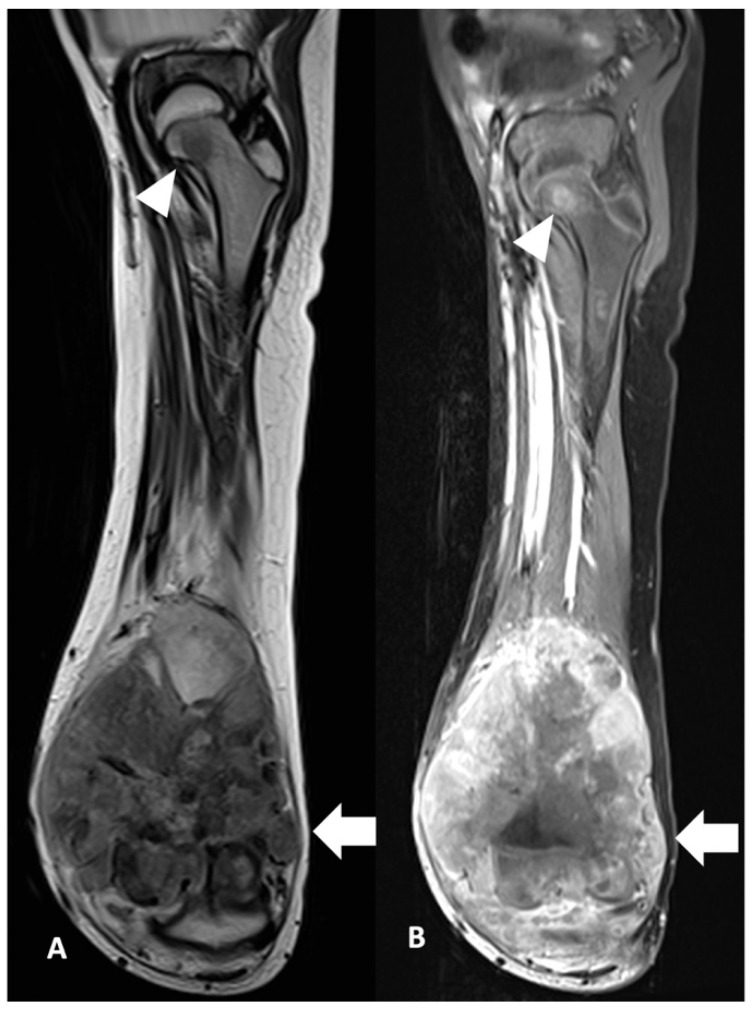
Skip metastasis. Coronal view of MRI left femur of a 10-year-old boy in (**A**) T2W and (**B**) T1FS CE show distal osteosarcoma (white arrow) with skip metastasis at the femoral neck (white arrowhead). The child had tumour progression post-treatment within 2 years of diagnosis.

Skip metastasis (SM) is consistently associated with poorer outcome (Figure 7). Conversely, the 5-year survival probability in a previous study performed by the Cooperative Osteosarcoma Study Group was 50% which was stated as possibly the result of successful surgery in their cohort. In their series, the rate of amputations was higher in patients with skips compared with osteosarcoma patients without skips and they suggested the role of limb salvage in improving survival rate among patients with SM [17]. A study undertaken in the UK evaluated whole-bone MRI in osteosarcoma and demonstrated 83.3% (n = 202) of patients had no SM and 39 (16.2%) of patients had SM. The presence of SM was significantly associated with both lung metastases (*p* < 0.001) and skeletal metastases (*p* < 0.001), but not with chemotherapy response (*p* = 0.24). Patients with SM also had poorer survival (*p* < 0.001) [18]. Failure of resection of a skip lesion is a cause of local recurrence and significantly contributes to the poor outcome [5].

Among the MRI features analysed, only tumour size and the presence of skip metastases showed a significant correlation with 2-year outcomes. Other factors such as fluid–fluid levels (FFL), concentric growth pattern and regional lymphadenopathy tended to show poorer 2-year outcome despite not being statistically significant, likely influenced by the limited sample size of this study.

FFLs typically appear as horizontal interfaces within cystic or necrotic areas of the tumor, often seen on T2-weighted MRI sequences. Previous studies suggest that the presence of FFL on initial MRI is a poor prognostic factor, indicating necrotic or haemorrhagic component due to a highly aggressive tumour. Once FFL is present within the tumour, the size of FFL appears to be inversely correlated with poor prognosis and degree of malignancy [19].

While FFLs can be observed in both benign and malignant bone lesions, in the context of pediatric osteosarcoma, their presence has been increasingly recognized as a potential prognostic indicator. A recent retrospective study found that FFLs were present in approximately 19% of patients with stage IIB osteosarcoma. Interestingly, both extensive and minimal FFLs were associated with poor treatment outcomes, including suboptimal histological response to neoadjuvant chemotherapy and lower overall survival rates. This suggests that FFLs may be a marker of tumor biology indicative of chemoresistance or aggressive tumor behavior. FFLs occupying less than one-third of the tumour have been associated with an unfavourable response to chemotherapy [9,19].

Published studies have reported a predominance of concentric over longitudinal tumour growth patterns, whereas this study found an equal distribution. One study identified a poorer prognosis in patients with concentric tumours, while another did not find a statistically significant association [8,20]. Similarly, our findings suggest that patients with concentric tumours may have worse outcomes, though this association did not reach statistical significance. The poorer prognosis of concentric tumours may be linked to their predominant location within the bone cortex and endosteal lining, regions considered chemoresistant zones, which could contribute to worse outcomes [8].

Regional lymph node involvement in osteosarcoma is rare but associated with poor prognosis. This rarity is due to the lack of lymphatic drainage in bone cortex [21]. In this study, patients with regional lymphadenopathy had poorer 2-year survival, though this was not statistically significant. The assessment of regional lymphadenopathy in MRI poses challenges, as metastatic nodes may be overreported. Differentiating malignant from reactive nodes is difficult, with size alone being unreliable. A previous study found that among four osteosarcoma patients with popliteal lymph nodes > 1 cm, only one was metastatic, with additional features of calcification and increased metabolic activity on PET scan. The author suggested that regional lymphadenopathy involvement should be suspected when the size exceeds 1 cm, show loss of the fatty hilum, contain calcifications, and/or exhibit metabolic activity on PET scans [22].

Previous studies have identified the humerus as a site associated with poorer prognosis in osteosarcoma. One study reported a 1.65 relative risk of poor five-year survival (*n* = 377, humerus = 36), while another found a 2.01 relative risk (*n* = 347, humerus = 32) [8,23]. However, this study showed a paradoxical 100% 2-year survival in proximal humerus osteosarcoma, which most likely contributed by small sample when compared with previous studies. This may also be due to the location itself as our population age match is not commonly obese and thus a lump or pain in the bone consultation by a doctor early.

Advanced MRI techniques such as diffusion weighted imaging (DWI) represent the movement of water molecules within tissues. Tumour tissue is expected to have high cellularity which restricts the free movement of water, whereas the non-packed cellular area such as necrotic tissue allows free movement of water. This physical property can be measured using the apparent diffusion coefficient (ADC), which may, therefore, be expected to increase in the setting of adequate treatment response. A similar principal was also applied to an MR perfusion study which is more established in brain tumour imaging that can be further explored for bone tumours in the future [5].

We acknowledge that this study was limited by the small sample size, with only 28 patients included, which reduces statistical power and may have contributed to the lack of significant correlation between MRI features and survival. Additionally, being a single-centre study, our findings may not be generalizable to broader populations, as institutional differences in imaging protocols, treatment strategies, and patient demographics could influence outcomes. A retrospective study is also subjected to missing data, inconsistencies in imaging techniques, and treatment approach which was the case in this study as more than half of our study population needed to be excluded. The variability in treatment protocols including differences in chemotherapy regimens could also have influenced the survival outcome, making it difficult to isolate the impact of MRI features.

These limitations highlight the need for larger, multicentre studies with standardized imaging and treatment protocols to provide a clearer understanding of the prognostic role of MRI features in osteosarcoma. A correlation with histopathological features could also provide a more definitive assessment of tumour aggressiveness and response to treatment.

## 5. Conclusions

In conclusion, this study demonstrated that larger tumour size (>300 mls) and skip metastases significantly correlate with poorer 2-year survival in osteosarcoma. While MRI features like fluid–fluid levels, concentric growth, physeal involvement, and regional lymphadenopathy has been associated with poor 2-year prognosis though not statistically significant.

## Figures and Tables

**Table 1:** MRI lower limb protocol for suspected bone tumour.

MRI Sequence	TR (ms)	TE (ms)	Slice Thickness (mm)	FOV * (cm)	Matrix
Coronal T1-weighted (T1W)	400	20	3	16–24	256 × 192
Sagittal T2-weighted (T2W)	3500	120	3	16–24	256 × 192
Axial T2 FS (fat sat)	4000	100	3	16–24	320 × 224
Coronal STIR	5000	50	3	16–26	256 × 192
Axial, coronal, and sagittal post-contrast T1 FS	500	20	3	16–24	256 × 192
DWI (diffusion weighted imaging)	4500	min (~50–100)	4	20–26	128 × 128
Axial and coronal 3D GRE/VIBE fat saturation	15	3	1 (isotropic voxels)	20–24	256 × 256
Coronal bilateral lower limb STIR	5000	50	5	40–48	256 × 192

* Field of View (FOV) must include both ends of the joint spaces.

**Table 2:** Baseline osteosarcoma patients’ demographics in the study population (*n* = 48).

		Number of Patient, *n* (%)
Sex	Male	29 (61.7%)
	Female	18 (38.3%)
Age (years)	Mean	17
	Min	6
	Max	62
Race	Malay	34 (72.3%)
	Chinese	6 (12.8%)
	Indian	1 (2.1%)
	Others ^1^	6 (12.8%)

^1^ Others—Foreigner (*n* = 5), Dayak (*n* = 1).

**Table 3:** Tumour characteristics and outcome.

MRI Features	Variables	Outcome		*p*-Value
		Good	Poor	
Tumour Volume (MLS)	<300 MLS	11 (73.3%)	4 (26.7%)	0.008 *
	>300 MLS	3 (23.1%)	10 (76.9%)	
Tumour Growth Pattern	Concentric	3 (30.0%)	7 (70.0%)	0.258
	Eccentric	6 (66.7%)	3 (33.3%)	
	Longitudinal	5 (55.6%)	4 (44.4%)	
	Concentric	3 (30.0%)	7 (70.0%)	0.115
	Eccentric + Longitudinal	11 (61.1%)	7 (38.9%)	
Tumour Location	Femur	7 (43.8%)	9 (56.2%)	0.310
	Tibia	3 (60.0%)	2 (40.0%)	
	Humerus	3 (100.0%)	0 (0.0%)	
	Fibula	1 (33.3%)	2 (66.7%)	
	Ilium	0 (0.0%)	1 (100.0%)	

* Statistically significant; *p* < 0.05.

**Table 4:** Pretreatment MRI features and 2-year outcome.

MRI Features	Variables	Outcome		*p*-Value
		Good	Poor	
Skip Metastasis	Presence	0 (0.0%)	5 (100.0%)	0.041 *
	Absence	14 (60.9%)	9 (39.1%)	
Features of FFL	Presence	5 (33.3%)	10 (66.7%)	0.058
	Absence	9 (69.2%)	4 (30.8%)	
	Present: <1/3	3 (33.3%)	6 (66.7%)	0.472
	Present: 1/3–2/3	0 (0.0%)	2 (100.0%)	
	Present: >2/3	2 (50.0%)	2 (50.0%)	
Pathological fracture	Presence	1 (50.0%)	1 (50.0%)	1.000
	Absence	13 (50.0%)	13 (50.0%)	
Neurovascular bundle involvement	Presence	8 (44.4%)	10 (55.6%)	0.430
	Absence	6 (60.0%)	4 (40.0%)	
Physeal involvement	Presence	10 (43.5%)	13 (56.5%)	0.098
	Absence	4 (100.0%)	0 (0.0%)	
Joint involvement	Presence	9 (47.4%)	10 (52.6%)	1.000
	Absence	5 (55.6%)	4 (44.4%)	
Regional lymphadenopathy	Presence	5 (35.7%)	9 (64.3%)	0.131
	Absence	9 (64.3%)	5 (35.7%)	

* Statistically significant; *p* < 0.05.

## Data Availability

Research data are stored in an institutional repository and will be shared upon request to the corresponding author.
